# Prevalence and molecular identification of *Borrelia* spirochetes in *Ixodes granulatus* ticks collected from *Rattus losea* on Kinmen Island of Taiwan

**DOI:** 10.1186/1756-3305-5-167

**Published:** 2012-08-10

**Authors:** Li-Lian Chao, Li-Ling Liu, Chien-Ming Shih

**Affiliations:** 1 Graduate Institute of Pathology and Parasitology, Department of Parasitology and Tropical Medicine, National Defense Medical Center, Taipei, Taiwan, Republic of China; 2 Research Center for Biotechnology and Medicine Policy, Taipei, Taiwan, Republic of China

**Keywords:** *Borrelia*, *Ixodes granulatus*, Tick, Genetic diversity, Taiwan

## Abstract

**Background:**

*Ixodes granulatus* is widely distributed in various countries of Southeast Asia and Taiwan. Although this tick species is presumed to be the vector for the enzoonotic transmission of *Borrelia* spirochetes in the Taiwan area, the prevalence of infection and genetic diversity of *Borrelia* spirochetes harbored by this tick species need to be further determined.

**Methods:**

DNA extraction was performed from individual tick specimens collected from *Rattus losea* on Kinmen Island of Taiwan. *Borrelia* infection in *I*. *granulatus* ticks was detected by performing a specific PCR assay based on the 5S-23S intergenic spacer amplicon gene of *B. burgdorferi* sensu lato. The genetic identities of detected spirochetes were identified by gene sequencing and phylogenetic analysis.

**Results:**

*Borrelia* infection was detected in nymph, male, and female stages of *Ixodes granulatus* ticks with an infection rate of 42.9%, 36%, and 52.7%, respectively. Genospecies identification reveals that *B. valaisiana* is the main genotype (70.7%) as compared to the genotype of *B. burgdorferi* sensu stricto (15.4%). Phylogenetic analysis revealed that these detected spirochetes were genetically affiliated to the genospecies *B. valaisiana* and *B. burgdorferi* sensu stricto, with a high sequence homology within the genospecies of *B. valaisiana* (95.8 to 100%) and *B. burgdorferi* sensu stricto (97.2 to 100%), respectively.

**Conclusions:**

This study highlights the significance of high prevalence and genetic diversity of *Borrelia* spirochetes in *I. granulatus* ticks collected from *Rattus losea* on Kinmen Island of Taiwan. Intraspecific analysis also revealed that *B. valaisiana* species detected in Kinmen Island can be easily distinguished from the European group of *B. valaisiana* and other genospecies of *Borrelia* spirochetes. This may imply an enzoonotic cycle between *I. granulatus* ticks and rodent hosts that maintains *Borrelia* spirochetes in Kinmen Island as well as Southeast Asia.

## Background

Lyme disease spirochetes, *Borrelia burgdorferi* sensu lato, was first identified within the gut of vector ticks [1] and the spirochete species can be classified into at least thirteen genospecies based on their genetic differences [2-5]. The tick species of *Ixodes ricinus* complex serve as the main vectors for transmission and perpetuation of *B. burgdorferi* spirochetes through a natural cycle between vector ticks and rodent hosts in North America and Europe [6,7]. Although *I. persulcatus* and *I. ovatus* have been recognized as the principle vector for the transmission of *B. burgdorferi* spirochetes in Northeast Asia, including the northeastern regions of China, Korea, and Japan [8-11], the hard ticks of *I. granulatusHaemaphysalis longicornis*, and *H*. *bispinosa* were suggested as the principle vectors for the transmission of *B. burgdorferi* spirochetes in the southwestern regions (adjacent to Taiwan) of China [12,13].

The abundance and widespread distribution of *I. granulatus* has been recorded for the first time from various countries in Southeast Asia and Taiwan [14]. The medical importance with the recent emergence of human babesiosis [15] and Lyme borreliosis [16] in Taiwan raises the focus of research attention on *I. granulatus* ticks. Indeed, Lyme disease spirochetes (*B. burgdorferi* sensu lato) have been isolated from six species of rodent hosts in Taiwan [17] and all these Taiwan isolates were genetically classified into the genospecies of *B. burgdorferi* sensu stricto [18,19]. In addition, *Borrelia* infection has also been detected in *I. granulatus* ticks collected from the Kinmen Island of Taiwan [20]. Although the hard tick of *I. granulatus* was presumed to be the tick vector for the enzoonotic transmission of *Borrelia* spirochetes in the Taiwan area [21], the prevalence of infection and genetic diversity of *Borrelia* spirochetes harbored by this tick species in Kinmen Island needs to be further defined.

The 5S (*rrf*)-23S (*rrl*) intergenic spacer amplicon gene is unique and highly conserved in *B*. *burgdorferi* sensu lato [22,23]. The diversity of this gene is useful for distinguishing the genetic heterogeneity among different *Borrelia* isolates [24-26]. Indeed, genetic identity of *Borrelia* spirochetes was clarified by analyzing the sequence homology of 5S (*rrf*)-23S (*rrl*) intergenic spacer amplicon genes of *B. burgdorferi* sensu lato isolated from various biological sources [2,13,27,28]. In addition, different genospecies of *B. burgdorferi* sensu lato are distributed unevently throughout the world and are associated with distinct ecologic features [2]. It may be that the *Borrelia* spirochetes that exist in *I. granulatus* ticks of Kinmen Island are genetically affiliated to the genospecies discovered in Asia, which are distinct from the *Borrelia* spirochetes within common vector ticks (*I. ricinus* complex) discovered in Europe and the United States. Thus, the objectives of the present study intend to determine the prevalence of *Borrelia* spirochetes within *I. granulatus* ticks by polymerase chain reaction (PCR) assay targeting the 5S (*rrf*)-23S (*rrl*) intergenic spacer amplicon gene of *B. burgdorferi* sensu lato and to clarify the genetic identity of detected spirochetes by analyzing phylogenetic relationships with other *Borrelia* species that have been documented in GenBank.

## Methods

### Collection and identification of tick specimens

**Figure 1:**
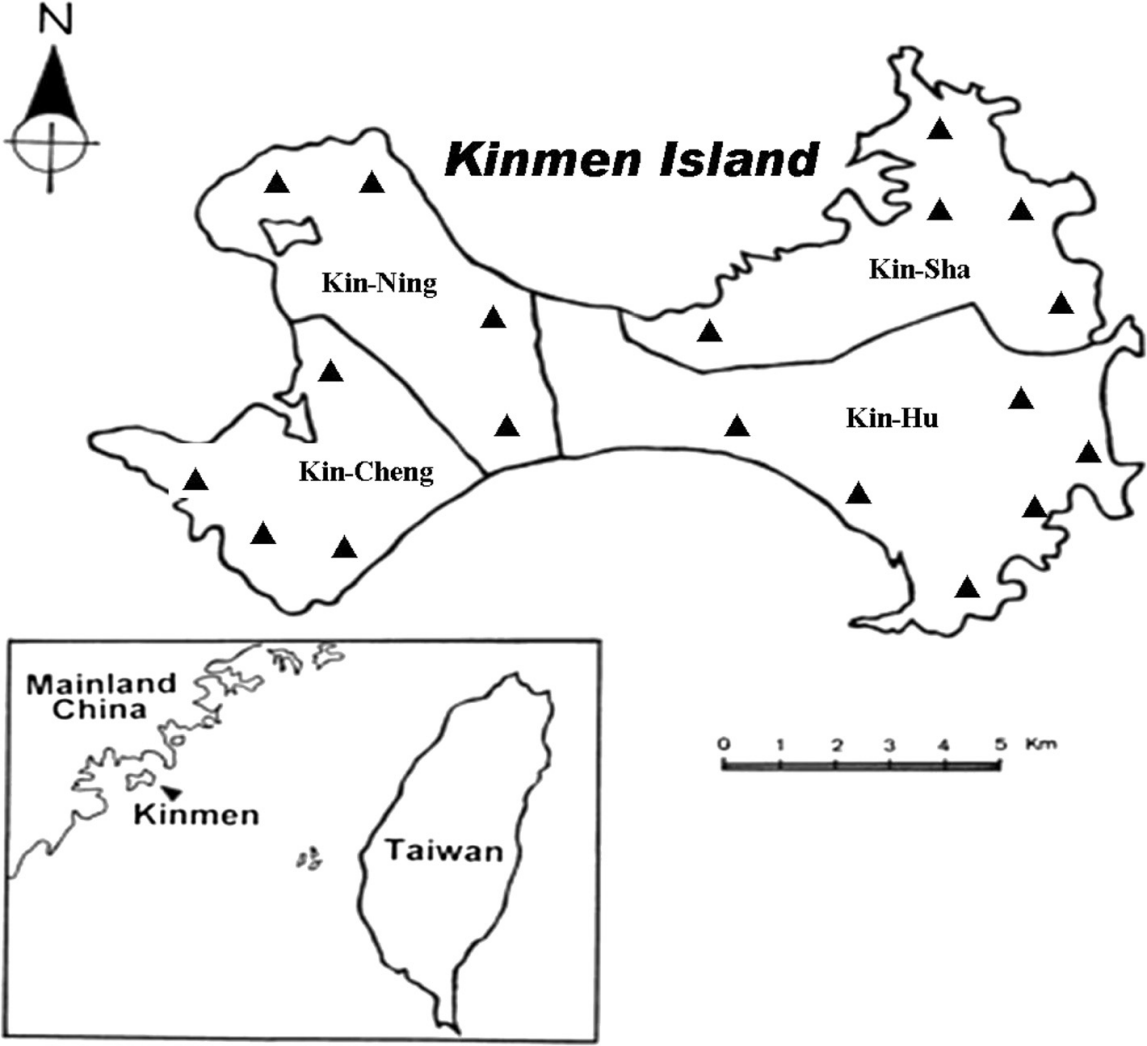
** Map of Taiwan and Kinmen Island, showing the geographic location of Kinmen Island and the collection sites (▴) for*I. granulatus*ticks removed from captured rodents.**

All specimens of adult and nymphal ticks were removed from rodents captured at various field sites of four townships in Kinmen Island (Figure 1). All field-collected ticks were subsequently stored in separate mesh-covered and plaster-bottomed vials. Adult and nymphal ticks of *I. granulatus* collected from Kinmen Island of Taiwan were identified to species level on the basis of their morphological characteristics, as described previously [29]. In addition, ultrastructural observations by scanning electron microscope (SEM) were also used to identify the morphological features of *I. granulatus* ticks, as described previously [29].

### DNA extraction from tick specimens

Total genomic DNA was extracted from individual tick specimens used in this study. Briefly, tick specimens were cleaned by sonication for 3–5 min in 75% ethanol and then washed twice in sterile distilled water. Afterwards, each individual tick specimen was dissected into pieces, placed in a microcentrifuge tube filled with 180-μl lysing buffer solution supplied in the DNeasy Blood & Tissue Kit (catalogue no. 69506, Qiagen, Hilden, Germany) and then homogenized with a sterile tissue grinder (catalogue no. 358103, Wheaton Scientific Products, Millville, NJ, USA). The homogenate was centrifuged at room temperature and the supernatant fluid was further processed using a Dneasy Blood & Tissue Kit, as per manufacturer’s instructions. After filtration, the filtrate was collected and the DNA concentration was determined spectrophotometrically with a DNA calculator (GeneQuant II; Pharmacia Biotech, Uppsala, Sweden).

### DNA amplification by polymerase chain reaction (PCR)

DNA samples extracted from the tick specimens were used as a template for PCR amplification. A nested PCR was performed with primers designed to amplify the variable spacer region between two conserved duplicate structures. A specific primer set corresponding to the 3’ end of the 5S rRNA (*rrf*) (5’-CGACCTTCTTCGCCTTAAAGC-3’) and the 5’ end of the 23S rRNA (*rrl*) (5’-TAAGCTGACTAATACTAATTACCC-3’) was designed and applied for the primary amplification, as described previously [23]. In the nested PCR, a primer set of primer 1 (5’-CTGCGAGTTCGCGGGAGA-3’) and primer 2 (5’-TCCTAGGCATTCACCATA-3’) was used and expected to yield a 226–266 bp fragment depending on the *Borrelia* strain, as described previously [30]. All PCR reagents and Taq polymerase were obtained and used as recommended by the supplier (Takara Shuzo Co., Ltd., Japan). Briefly, a total of 0.2-μmol of the appropriate primer set and various amounts of template DNA (0.1-0.3 μg) were used in each 50-μl reaction mixture. The PCR amplification was performed with a Perkin-Elmer Cetus thermocycler (GeneAmp system 9700; Applied Biosystems, Taipei, Taiwan), and the primary amplification included 2 min denaturation at 96°C followed by 30 cycles of the following conditions: denaturation at 94°C for 30 s, annealing at 55°C for 30 s, and extension at 72°C for 40 s. Nested amplification was performed under the same conditions, except for annealing at 59°C for 30 s. Thereafter, amplified DNA products were electrophoresed on 2% agarose gels in Tris-Borate-EDTA (TBE) buffer and visualized under ultraviolet (UV) light after staining with ethidium bromide. A DNA ladder (1-kb plus, catalogue no. 10787–018, Gibco BRL, Taipei, Taiwan) was used as the standard marker for comparison. A negative control of distilled water was included in parallel with each amplification.

### Sequence alignments and phylogenetic analysis

After purification with a QIAquick PCR purification kit (catalogue no. 28104, Qiagen, Hilden, Germany), sequencing reactions were performed with 25 cycles under the same conditions and same primer set (primer 1 and primer 2) of nested amplification by using the Big Dye Terminator Cycle Sequencing Kit (V3.1) under an ABI Prism 377–96 DNA sequencer (Applied Biosystems, Foster City, CA, USA). The resulting sequences (241–245 bp) were initially edited by BioEdit software (V5.3) and aligned with the CLUSTAL W software [31]. Afterwards, the aligned sequences (205–209 bp) were further analyzed by comparing with other *Borrelia* sequences based on the type-strain of different genospecies and different geographic origin of *Borrelia* spirochetes that were available in GenBank. Phylogenetic analysis was performed by neighbour-joining (NJ) compared with maximum parsimony (MP) methods to estimate the phylogeny of the entire alignment using MEGA 4.0 software [32]. A similarity matrix was constructed using DNASTAR program (Lasergene, version 8.0). All phylogenetic trees were constructed and performed with 1000 bootstrap replications to evaluate the reliability of the constructions, as described previously [33].

### Nucleotide sequence accession numbers

The nucleotide sequences of PCR-amplified 5S (*rrf*)-23S (*rrl*) intergenic spacer amplicon genes of *Borrelia* spirochetes determined in this study have been registered and assigned the following GenBank accession numbers: strains KC-44 (JF970243), KH-05 (JF970244), KH-13 (JF970245), KH-74 (JF970246), KN-11 (JF970247), KS-61 (JF970248), KS-62 (JF970249), KH-58 (JF970250), KC-14 (JF970251), KC-49 (JF970252), KH-70 (JF970253), KH-71 (JF970254), KS-18 (JF970255), and KS-39 (JF970256). For phylogenetic analysis, nucleotide sequences of 5S (*rrf*)-23S (*rrl*) intergenic spacer amplicon genes from another 19 strains of *Borrelia* species were included for comparison (Table 1).

**Table 1:** **Genospecies and strains of*Borrelia*spirochetes used for analysis in this study**

**Genospecies and strain**	**Origin of*Borrelia*strain**		**5S (*rrf*)-23S (*rrl*) gene accession number**^**a**^
	**Biological**	**Geographic**	
Taiwan strains			
KC-44	*Ixodes granulatus*	Kin-Cheng, Kinmen	**JF970243**
KH-05	*I. granulatus*	Kin-Hu, Kinmen	**JF970244**
KH-13	*I. granulatus*	Kin-Hu, Kinmen	**JF970245**
KH-74	*I. granulatus*	Kin-Hu, Kinmen	**JF970246**
KN-11	*I. granulatus*	Kin-Ning, Kinmen	**JF970247**
KS-61	*I. granulatus*	Kin-Sha, Kinmen	**JF970248**
KS-62	*I. granulatus*	Kin-Sha, Kinmen	**JF970249**
KH-58	*I. granulatus*	Kin-Hu, Kinmen	**JF970250**
KC-14	*I. granulatus*	Kin-Cheng, Kinmen	**JF970251**
KC-49	*I. granulatus*	Kin-Cheng, Kinmen	**JF970252**
KH-70	*I. granulatus*	Kin-Hu, Kinmen	**JF970253**
KH-71	*I. granulatus*	Kin-Hu, Kinmen	**JF970254**
KS-18	*I. granulatus*	Kin-Sha, Kinmen	**JF970255**
*B. valaisiana*			
KS-39	*I. granulatus*	Kin-Sha, Kinmen	JF970256
VS116	*I. ricinus*	Switzerland	L30134
UK	*I. ricinus*	England	L30133
QLZSP1	*I. granulatus*	China	EU247839
QTDM2	*I. granulatus*	China	EU429347
CKA2a	*Apodemus agrarius*	China	AB022124
OG1/01	*I. granulatus*	Japan	AB091441
OG45/01	*I. granulatus*	Japan	AB091455
HN6	*I. granulatus*	Korea	AF058705
*B. burgdorferi* sensu stricto			
B31	*I. scapularis*	USA	L30127
JD1	*I. scapularis*	USA	AY032911
TWKM5	*Rattus norvegicus*	Taiwan	AY032908
*B. garinii*			
20047	*I. ricinus*	France	L30119
NP81	*I. persulcatus*	Japan	D84406
*B. afzelii*			
VS461	*I. ricinus*	Switzerland	L30135
PGau	Human skin	Germany	DQ111066
*B. bissettii*			
DN127	*I. pacificus*	USA	L30126
CA376	*Neotoma fuscipes*	USA	AY177634
*B. sinica*			
CMN1a	*Niviventer sp.*	China	AB022129
CMN3	*Niviventer sp.*	China	AB022131

^a^GenBank accession numbers (**JF970243 ~ JF970256**) were submitted by this study.

## Results

### PCR detection of *Borrelia* infection in *I. granulatus* ticks

To verify the existence of *Borrelia* spirochetes in *I. granulatus* ticks removed from rodents of Kinmen Island. A total of 292 field rodents (*Rattus losea*) were captured and examined for *I. granulatus* ticks from four townships of Kinmen Island. An overall infestation was observed on 62% (181/292) captured rodents with an average density of 1.44 ticks per infested rodent. A total of 261 ticks (131 female, 25 male, and 105 nymph) were examined and tested for the evidence of spirochete infection by PCR using specific primers targeting the 5S (*rrf*)-23S (*rrl*) intergenic spacer amplicon genes of *B. burgdorferi* sensu lato. Results indicate that *Borrelia* infections were detected in 52.7% (69/131) of females, 36% (9/25) of males, and 42.9% (45/105) of nymphs of *I. granulatus* ticks (Table 2). The highest infection was detected in 52% (65/125) of *I. granulatus* ticks collected from Kin-Hu township. In contrast, the lowest infection was detected in 12.5% (1/8) of *I. granulatus* ticks collected from Kin-Ning township. The overall infection was detected in 47.1% (123/261) of *I. granulatus* ticks collected from Kinmen Island.

**Table 2:** **Detection of*Borrelia*infection in various stages of*I. granulatus*ticks collected from four townships of Kinmen Island by PCR assay targeting the 5S (*rrf*)-23S (*rrl*) intergenic spacer gene of*B. burgdorferi*sensu lato**

**Township**	**Stage of tick**^**a**^			**Total No. infected/No. tested (%)**
	**Male**	**Female**	**Nymph**	
	**No. infected/No. tested (%)**	**No. infected/No. tested (%)**	**No. infected/No. tested (%)**	
Kin-Hu	6/14 (42.9)	40/65 (61.5)	19/46 (41.3)	65/125 (52.0)
Kin-Sha	3/9 (33.3)	25/57 (43.9)	19/36 (52.8)	47/102 (46.1)
Kin-Ning	0/0 (0.0)	1/3 (33.3)	0/5 (0.0)	1/8 (12.5)
Kin-Cheng	0/2 (0.0)	3/6 (50.0)	7/18 (38.9)	10/26 (38.5)
Total	9/25 (36.0)	69/131 (52.7)	45/105 (42.9)	123/261 (47.1)

^a^All *I. granulatus* ticks were removed from the rodent host of *Rattus losea*.

### Genetic identification of detected spirochetes

To clarify the genetic identity of *Borrelia* spirochetes detected in *I. granulatus* ticks collected from Kinmen Island, sequences of PCR-amplified 5S (*rrf*)-23S (*rrl*) intergenic spacer fragments of 123 strains of *Borrelia* spirochetes were aligned and compared with the downloaded sequences of known genospecies of *Borrelia* spirochetes. Results indicate that *B. valaisiana* was detected as the main genotype in 70.7% (87/123) of *I. granulatus* ticks and the genotype of *B. burgdorferi* sensu stricto was also detected in 15.4% (19/123) of *I. granulatus* ticks (Table 3). However, there still are 13.8% (17/123) of untyped strains.

**Table 3:** **Genospecies identification of*Borrelia*spirochetes detected in*I. granulatus*ticks collected from four townships of Kinmen Island, Taiwan**

**Township**	**Positive No. by PCR**	**Genospecies determined by 5S (*rrf*)-23S (*rrl*) gene sequences**		
		***B. burgdorferi* sensu stricto**	***B. valaisiana***	**Untyping**
Kin-Hu	65	12	46	7
Kin-Sha	47	4	35	8
Kin-Ning	1	1	0	0
Kin-Cheng	10	2	6	2
Total (%)	123	19 (15.4)	87 (70.7)	17 (13.8)

### Sequence analysis of detected spirochetes

Sequence similarity of PCR-amplified 5S (*rrf*)-23S (*rrl*) intergenic spacer fragments of 14 selected strains of *Borrelia* spirochetes from Kinmen Island were aligned and compared with the downloaded sequences of another 19 strains of *Borrelia* spirochetes (8 *B. valaisiana*, 3 *B. burgdorferi* sensu stricto, 2 *B. garinii*, 2 *B. afzelii*, 2 *B. bissettii*, and 2 *B. sinica*) from GenBank. The nucleotide sequences between the 14 *Borrelia* spirochetes of Kinmen Island are highly homogeneous with a high sequence homology within the genospecies of *B. burgdorferi* sensu stricto (97.2 to 100%) and *B. valaisiana* (95.8 to 100%), respectively (Table 4). However, intraspecific analysis based on the sequence similarity reveals that all these *B. valaisiana* spirochetes (GenBank accession numbers: JF970243, JF970246, JF970248-9, and JF970251-6) of Kinmen Island are closely related with the Asian group (China, Japan, and Korea) of *B. valaisiana* (GenBank accession numbers: EU247839, EU429347, AB022124, AB091441, AB091455, and AF058705) and can be distinguished from the European group (Switzerland and England) of *B. valaisiana* (GenBank accession numbers: L30133 and L30134), as well as other genospecies of *B. burgdorferi* sensu lato (Table 4).

**Table 4:** **Sequence similarity between 5S (*rrf*)-23S (*rrl*) gene sequences from Taiwan strains of*Borrelia*detected in*Ixodes granulatus*ticks and strains of other genospecies of*Borrelia***

**Genospecies and strain**^**a**^	**B31**	**TWKM5**	**KH5**	**KN11**	**KH13**	**UK**	**OG1**	**HN6**	**QLZSP1**	**KC44**	**KH74**	**KS61**	**KS62**	**20047**	**VS461**	**DN127**	**CMN1a**
*Bb*ss B31	-	99.1	99.1	97.2	97.7	89.3	90.7	90.7	90.7	90.7	90.2	90.7	90.2	93.0	86.0	92.1	81.8
*Bb*ss TWKM5		-	100	98.1	98.6	90.2	90.7	90.7	90.7	90.7	90.2	90.7	90.2	93.0	86.0	92.1	81.8
KH5			-	98.1	98.6	90.2	90.7	90.7	90.7	90.7	90.2	90.7	90.2	93.0	86.0	92.1	81.8
KN11				-	98.6	89.3	89.7	89.7	89.7	89.7	89.3	89.7	89.3	92.1	85.5	90.2	79.9
KH13					-	88.8	89.3	89.3	89.3	89.3	88.8	89.3	88.8	91.6	84.6	90.7	81.3
*Bv* UK						-	95.8	95.8	95.8	95.8	96.3	95.8	95.8	93.0	87.4	88.3	80.8
*Bv* OG1							-	100	100	99.1	99.5	99.1	99.1	93.5	88.3	87.9	80.4
*Bv* HN6								-	100	99.1	99.5	99.1	99.1	93.5	88.3	87.9	80.4
*Bv* QLZSP1									-	99.1	99.5	99.1	99.1	93.5	88.3	87.9	80.4
KC44										-	99.5	100	99.1	93.5	88.3	88.8	81.3
KH74											-	99.5	99.5	93.0	87.9	88.3	80.8
KS61												-	99.1	93.5	88.3	88.8	81.3
KS62													-	93.0	87.9	88.3	80.8
*Bg* 20047														-	90.7	90.7	82.7
*Ba* VS461															-	85.5	82.7
*Bbis* DN127																-	79.9
*Bs* CMN1a																	-

^a^Strains: B31 and TWKM5, *B. burgdorferi* sensu stricto (*Bbss*); UK, OG1, HN6 and QLZSP1, *B. valaisiana* (*Bv*); 20047, *B. garinii* (*Bg*); VS461, *B. afzelii* (*Ba*); DN127, *B. bissettii* (*Bbis*); CMN1a, *B. sinica* (*Bs*).

### Phylogenetic analysis of detected spirochetes

**Figure 2:**
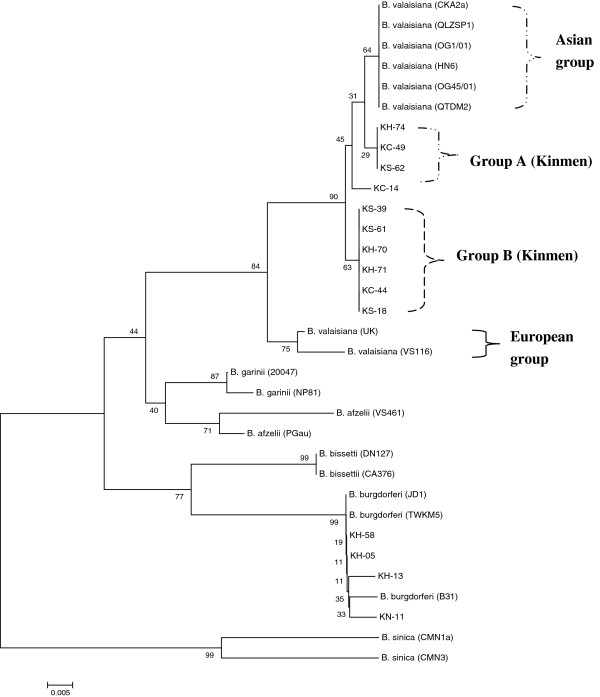
** Phylogenetic relationships based on the aligned sequences (205–209 bp) of 5S (*rrf*)-23S (*rrl*) rRNA gene were used to compare the genetic variation between 14*Borrelia*spirochetes detected in*I. granulatus*ticks from Kinmen Island of Taiwan and 19 other strains of*Borrelia*spirochetes.** The tree was constructed and analyzed by the neighbour-joining method using 1000 bootstrap replicates. Numbers at the nodes indicate the percentages of reliability of each branch of the tree. Branch lengths are drawn proportional to the estimated sequence divergence.

Phylogenetic relationships based on the alignment of 5S (*rrf*)-23S (*rrl*) intergenic spacer sequences were performed to analyze the genetic divergence among 33 *Borrelia* spirochetes investigated in this study. Bootstrap analysis was used to analyze the repeatability of the clustering of specimens represented in phylogenetic trees. Phylogenetic trees constructed by both NJ (Figure 2) and MP (data not shown) analyses showed congruent basal topologies with nine major branches of distinguished clades. All *Borrelia* spirochetes detected in *I. granulatus* ticks from Kinmen Island represent two major groups of *B. valaisiana* spirochetes (groups A-B) which constituted a separate clade that can be easily distinguished from the European group of *B. valaisiana* spirochetes, and one major group of *Borrelia* spirochetes which was highly affiliated with the main genospecies of *B. burgdorferi* sensu stricto (strains B31, JD1, and TWKM5) (Figure 2). The phylogenetic analysis of NJ tree strongly supports the separation of different lineages between the *Borrelia* spirochetes from Kinmen Island and Europe with a bootstrap value of 84. These results reveal a lower genetic divergence within the same genospecies of *Borrelia* spirochetes from Kinmen Island of Taiwan, but a higher genetic variation among different genospecies or variant geographic origins of *Borrelia* spirochetes.

## Discussion

This study describes the first survey regarding the prevalence and genetic identification of *Borrelia* spirochetes detected in various stages of *I. granulatus* ticks collected from the offshore Kinmen Island of Taiwan. In our previous investigations, *B. burgdorferi* spirochetes had been isolated from six species of rodent hosts captured at various locations in Taiwan and *I. granulatus* ticks were observed on four species of highly infested rodent hosts [17]. Although the existence of zoonotic transmission of *Borrelia* spirochetes was suggested, the identification of *Borrelia* spirochetes within possible vector tick is required to verify the natural infection in Kinmen Island. Indeed, results from the present study confirm the high prevalence of *B. burgdorferi* sensu stricto and *B. valaisiana* spirochetes detected in various stages of *I. granulatus* ticks (Tables 2 and 3), and reveal that the rodent species of *R. losea* serves as the major infested host for maintaining the natural transmission of *Borrelia* spirochetes in Kinmen Island of Taiwan. Further investigations focusing on the seasonal abundance and prevalence of spirochetal infections among reservoir hosts would help to elucidate the enzoonotic transmission of *Borrelia* spirochetes in Kinmen Island of Taiwan.

The transmission cycle for *Borrelia* spirochetes in Southeast Asia remains elusive. It is assumed that different genospecies of *Borrelia* spirochetes is relevant to the distinct reservoir hosts and vector ticks [2]. Indeed, *B. valaisiana* has been isolated or detected from *I. ricinus* ticks and avain reservoirs from at least eight European countries [34-39]. In addition, *B. valaisiana*-related spirochetes were isolated mainly from rodent hosts and detected in various hard ticks (*I. nipponensisI. columnaeI. granulatus*, and *Haemaphysalis longicornis*) in Northeast Asia and Southwestern China [13,40-42]. However, *I. granulatus* is widespread in various countries of Southeast Asia and Taiwan [14]. Results from this study also verify the high prevalence of *B. valaisiana* and *B. burgdorferi* sensu stricto detected in *I. granulatus* ticks that are infested on the rodent host of *R. losea* in Kinmen Island. These observations may suggest that an enzoonotic cycle between rodent hosts and *I. granulatus* ticks exists, therefore perpetuating *Borrelia* spirochetes in Kinmen Island and Southeast Asia.

The existence of two tandemly duplicated copies of 5S (*rrf*)-23S (*rrl*) intergenic spacer genes in *B. burgdorferi* spirochetes is unique and has not been found in other eubacteria [22,23]. Taking advantage of this unique genomic character, the genetic identity of *Borrelia* spirochetes can be distinguished by their differential reactivities with genospecies-specific PCR primers targeting the 5S (*rrf*)-23S (*rrl*) intergenic spacer amplicon gene. Indeed, genetic heterogeneity can be further classified among *Borrelia* isolates that were previously identified as the same genospecies of atypical strains of *Borrelia* spirochetes [25,26]. Results from the present study also verify that the genetic identities of *Borrelia* spirochetes detected within *I. granulatus* ticks of Kinmen Island are highly homogeneous within the genospecies of *B. burgdorferi* sensu stricto and *B. valaisiana*, and were clearly distinguished from other genospecies of *Borrelia* spirochetes (Tables 2,3,4). Further application of these genospecies-specific PCR tools to analyze the 5S (*rrf*)-23S (*rrl*) genes of *Borrelia* spirochetes detected in various tick species would help to clarify the genetic divergence of *Borrelia* spirochetes transmitted in the natural cycle of Kinmen Island.

Phylogenetic relationships among *Borrelia* spirochetes can be constructed and determined by analyzing their sequence homogeneity of a specific target gene. Indeed, the sequence analysis of 5S (*rrf*)-23S (*rrl*) intergenic spacer amplicon gene among various *Borrelia* spirochetes had been proved useful to evaluate the taxonomic relatedness of *Borrelia* spirochetes derived from various biological and geographical sources [24,28,43,44]. Although PCR amplification of the intergenic spacer region located between the *rrf* and *rrl* genes of *B. burgdorferi* sensu lato had been reported to generate a DNA fragment of approximately 226–266 bp long [30], the variation of nucleotide sequence depends on the strain or group diversity of *Borrelia* spirochetes and may actually represent the genetic distance of phylogenetic divergence between or within the genospecies of *Borrelia* spirochetes [2,30,44]. In this study, phylogenetic analysis based on the sequences of 5S (*rrf*)-23S (*rrl*) intergenic spacer amplicon gene of *Borrelia* spirochetes from Kinmen Island demonstrated a high sequence homogeneity among *Borrelia* spirochetes within the genospecies of *B. burgdorferi* sensu stricto (Table 4; Figure 2). However, a high genetic heterogeneity within the genospecies of *B. valaisiana* spirochetes was also observed between the Asian group and European group of *Borrelia* strains (Figure 2). Although a low intraspecific variation was observed among the same Asian groups of *B. valaisiana*, all strains of *B. valaisiana* from Kinmen Island represented as two separate clades (groups A-B) that can be separated from the Asian group and clearly distinguished from the European group of *B. valaisiana* (Figure 2). The phylogenetic trees constructed by either NJ or MP analysis strongly support the discrimination recognizing the separation of different lineages of *B. valaisiana* detected from the Asian group, European group, and Kinmen Island of Taiwan. Accordingly, these observations reveal that all these *B. valaisiana* spirochetes detected in *I. granulatus* ticks from Kinmen Island represent two major groups forming a unique clade distinct from the European group of *B. valaisiana*.

## Conclusions

This study provides the first survey regarding the prevalence and genetic diversity of *Borrelia* spirochetes within *I. granulatus* ticks collected from Kinmen Island of Taiwan. Further application of this molecular tool to investigate the genetic variability among *Borrelia* spirochetes detected in different vector ticks and reservoir hosts may facilitate our understanding of the significance of genetic diversity in relation to the epidemiological features of *Borrelia* spirochetes in Southeast Asia.

## Competing interests

The authors declare that they have no competing interests.

## Authors’ contributions

CMS designed the study, and contributed with tick collection, data analysis, interpretation, and manuscript writing. LLC contributed with tick collection, tick identification, sequencing, and phylogenetic analysis. LLL performed DNA extraction, PCR amplification, and gel electrophoresis. All authors read and approved the final version of the manuscript.
